# MiR-199a-5p regulates sirtuin1 and PI3K in the rat hippocampus with intrauterine growth restriction

**DOI:** 10.1038/s41598-018-32189-5

**Published:** 2018-09-14

**Authors:** Juncao Chen, Xiaoyun Gong, Li Huang, Pingyang Chen, Tao Wang, Wei Zhou, Kaiju Luo, Jing Wang

**Affiliations:** 1 Institute of Pediatrics, Guangzhou Women and Children’s Medical Centre, Guangzhou Medical University, Guangzhou, 510623 China; 2 Division of Neonatology, The Second Xiangya Hospital, Central South University, 139 Renmin Middle Rd, Changsha, Hunan 410011 China; 3 Division of Neonatology, Guangzhou Women and Children’s Medical Centre, Guangzhou Medical University, Guangzhou, 510623 China

## Abstract

In humans, malnutrition during pregnancy results in intrauterine growth restriction (IUGR) and an increased risk of neurological morbidities; altered miRNA characteristics have been suggested to contribute to IUGR neurological pathogenesis. A miRNA microarray was used to identify differentially expressed miRNA molecules in the hippocampi of rats with IUGR. Five of the molecules in question were selectively validated using real-time PCR in rats with IUGR. We then investigated the role of miR-199a-5p in hippocampal pathology. Bioinformatics analysis results suggested that TNF-α, caspase-3 and SIRT1 were potential targets of miR-199a-5p. Changes in PI3K, SIRT1 and caspase-3 protein expressions levels in the hippocampus were confirmed by Western blot analysis (all *P* < 0.05). Studies using the pheochromocytoma cell line PC12 cells and primary neurons demonstrated that miR-199a-5p modulated PI3K, caspase-3 and SIRT1 expression. Additionally, there was an inverse correlation between miR-199a-5p and caspase-3 expression, though dual-luciferase reporter assays showed that caspase-3 is not a target of miR-199a-5p. We conclude that IUGR affects hippocampal miRNAs characteristics. Our results also indicated that aberrantly high expression levels of miR-199a-5p may play an important role in the pathogenesis of IUGR by regulating SIRT1 and PI3K.

## Introduction

Foetal development, which represents a critical period, can have major ramifications not only on the proper growth and development of the foetus but also on risk for disease later in life. A relevant marker of this phenomenon is intrauterine growth restriction (IUGR)^1^. Foetuses with IUGR are at an increased risk for both perinatal and long-term neurological morbidities, including decreased academic performance and standardized testing levels, which are tied to hippocampal functions. Previous studies have demonstrated that IUGR also reduces infant hippocampal volumes, a change that is associated with behavioural differences and learning disorders during childhood^2–4^.

An altered intrauterine environment can affect offspring gene expression through epigenetic mechanisms. The causes of brain modifications resulting from IUGR have been broadly categorized and include epigenetic modifications and other mechanisms. One epigenetic mode of alteration may result from aberrant microRNA (miRNA) expression. miRNAs are approximately 21–25-nucleotide long evolutionarily conserved non-coding RNAs that function as endogenous regulators of post-transcriptional gene expression. These small RNAs are capable of controlling gene expression by mediating mRNA degradation or translation inhibition^5^. In recent years, miRNAs have been shown to play important roles in a variety of physiological and pathological processes in humans and animals. For example, miR-199a may be involved in the pathophysiology of alcoholic liver disease and epilepsy by regulating hypoxia-inducible factor-1 alpha^6,7^. Previous studies have demonstrated that miRNAs play important roles in IUGR. MiR-141 was significantly up-regulated in both the placental tissues and plasma of women whose babies experienced IUGR and plays an important role in the pathogenesis of IUGR by suppressing pleomorphic adenoma gene 1^8,9^. Additionally, Maccani *et al*.^10^ demonstrated that decreased miR-16 and miR-21 expression levels in the placenta were significantly associated with low birth weight.

The primary goal of our study was to identify the hippocampal expression profiles of miRNAs that differed significantly between rats with IUGR and normal rats. In this study, miRNA expression profiles were investigated in the hippocampi of rats with IUGR using miRNA chips. The target genes of the differentially expressed miRNAs were predicted using bioinformatics methods, and a global analysis of the biological pathways and genes regulated by these miRNAs was performed.

## Results

### Brain weight and body weight were decreased in the IUGR group

In this study, the dams were fed with a 10% low-protein diet during pregnancy, and we selected offspring according to IUGR standards (the criterion is that a significant reduction in the foetal growth rate that results in a birth weight in the lowest 10th percentile for gestational age). Experimental samples were harvested at 24 h and on day 7, 21, and 56.

The birth weights of offspring whose mothers received a restricted-protein diet (IUGR group) met the criteria for IUGR (5.37 ± 0.46 g vs. 6.72 ± 0.49 g, the proportion of females and males in the two groups was 14:16 vs. 15:16). The rats with IUGR had mean brain weights that were 18% less than the brain weights of the control rats on DOL1 (day of life 1) and 11% less than the brain weights of the control rats on DOL7. However, the brain weights were similar between the two groups on both DOL21 and DOL56 (*P* *>* 0.05) (Fig. S1).

### Different miRNA expression levels in the hippocampus were noted between the two groups

To explore the hippocampal expression profiles of miRNAs that differed significantly between rats with IUGR and normal rats, a miRNA microarray was used to identify differentially expressed miRNA molecules in the hippocampi of rats with IUGR.

The miRNA expression levels in the IUGR group were compared with those in the control group on DOL7, and the levels of 27 miRNAs were significantly different (the signal intensity ratio of the IUGR/control group was either >2 or <0.5) [Table 1]. Compared with miRNAs in the control group, 18 of these miRNAs were up-regulated in the IUGR group, whereas 9 miRNAs were significantly down-regulated (the ratio was <0.5) [Table 1].

**Table 1:** The significantly differentially expressed miRNAs in normal and IUGR hippocampus at DOL7.

Upregulated miRNA Name	Fold Change (IUGR/control)	Downregulated miRNA Name	Fold Change (IUGR/control)
rno-miR-199a-5p	0.24	rno-miR-34b	3.85
rno-miR-325-3p	0.24	rno-miR-196b*	3.70
rno-miR-219	0.25	rno-miR-449a	3.57
rno-miR-664-1*	0.30	rno-miR-673	3.30
rno-miR-551b	0.32	rno-miR-204*	3.12
rno-miR-34C	0.32	rno-miR-702-5p	2.94
rno-miR-199a-3p	0.35	rno-miR-32*	2.70
rno-miR-873	0.41	rmo-mir-375	2.63
rno-miR-195*	0.47	rno-miR-872*	2.56
rno-miR-204	5.88	rno-miR-448	2.56
rno-miR-3594-3p	5.00	rno-miR-218b	2.17
rno-miR-483*	4.76	rno-miR-541*	2.13
rno-miR-322	4.35	rno-miR-487b	2.00
rno-miR-329*	4.17		

### Confirmation of the differentially expressed miRNAs in the hippocampus

**Figure 1:**
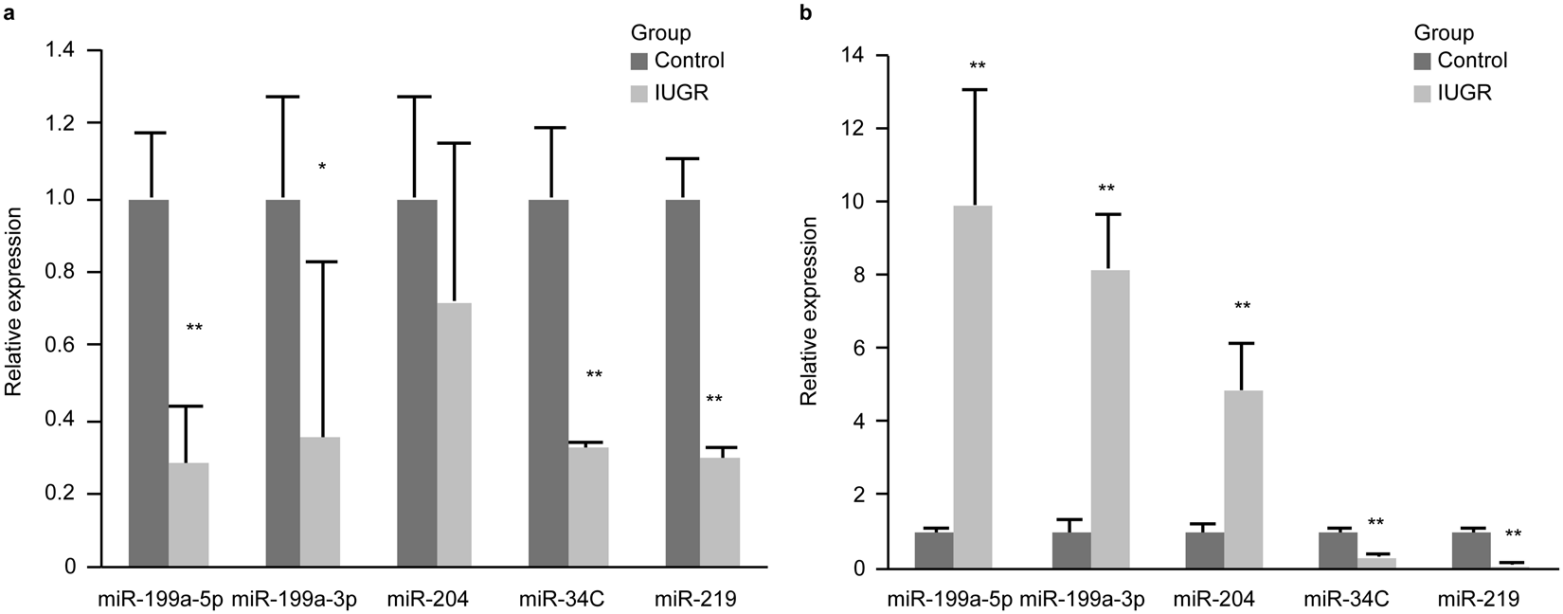
MiR-199a-3p, miR-199a-5p, miR-204,miR-34C and miR-219 expression levels in the hippocampi in the two groups on DOL 1 and DOL7 as measured via qRT-PCR. (**a**) miR-199a-3p, miR-199a-5p, miR-204, miR-34C and miR-219 expression levels between the control and IUGR groups on DOL7 (all groups, n = 8 rats). (**b**) The fold differences between the control and IUGR groups on DOL1. The values are expressed as the mean ± SD. *P < 0.05, **P < 0.01.

To determine the reliability of the miRNA microarray results, five miRNAs (miR-199a-3p, miR-199a-5p, miR-204, miR-219 and miR-34C) were selected for additional analysis via qRT-PCR); these miRNAs have been shown to be closely related to IUGR pathogeny. On DOL1, miR-204, miR-199a-3p, and miR-199a-5p expression levels were up-regulated more than 3-fold in the IUGR group compared with the expression in the control group (4.8-, 8.1-, and 8.9-fold, respectively). In contrast,miR-219 and miR-34C expression levels were significantly lower in the IUGR group than in the control group (3- and 8-fold, respectively) [Fig. 1b]. On DOL7, miR-219, miR-34C, miR-199a-3p, and miR-199a-5p expression levels were up-regulated more than 2-fold in the control group compared with the expression levels in the IUGR group (3-, 3.4-, 2.8- and 3.6-fold, respectively); however, miR-204 expression levels did not significantly change between the two groups (*P* > 0.05) [Fig. 1a].

### miRNA target prediction

To explore the potential mechanisms by which key miRNAs execute their functions in IUGR, three algorithms (miRwalk, TargetScan, and miRanda) were used to further analyse their target genes [Table S1].

**Figure 2:**
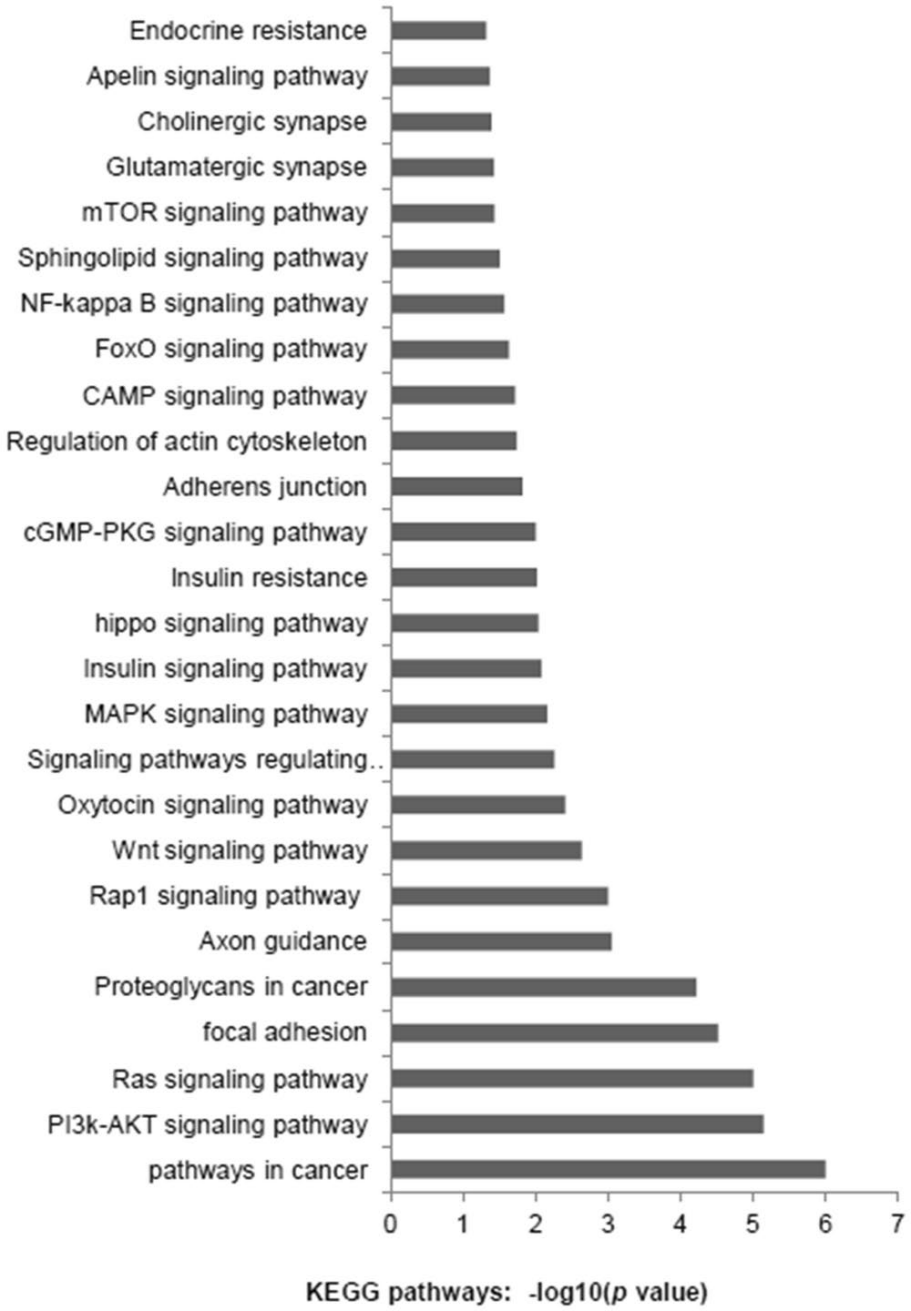
KEGG pathway analysis of differentially expressed miRNAs.

To identify the enriched pathways, Kyoto Encyclopaedia of Genes and Genomes pathway (KEGG) pathway analyses were performed by using DAVID functional annotation; then we confirmed 26 pathways (*P* < *0*.*05*) [Fig. 2]. We used PubMed with the search term “Intrauterine growth restriction”; the resulting article show that the apoptosis, PI3K- Akt/mTOR and insulin signalling pathways have been shown to play important roles in the pathogenesis of IUGR.

Additionally, some miRNAs shared common target genes that had a higher probability of being suppressed by miRNAs. For example, miR-199a-5p, miR-204 and miR-449a share the common target gene sirtuin1 (SIRT1), while miR-199a-5p, miR-204 and miR-448 have the common target gene caspase-3 (Casp3). The phosphoinositide-3-kinase regulatory subunit 1 (PI3K) gene is a common target gene for miR-32 and miR-322, while the tumour necrosis factor-α (TNF-α) gene is a common target gene for both miR-199a-5p and miR-204.

According to above principles, we explored the expression of SIRT1, Casp3, PI3K and TNF-α in the two groups.

### TNF-α, SIRT1, PI3K and Casp3 were altered in the IUGR group

**Figure 3:**
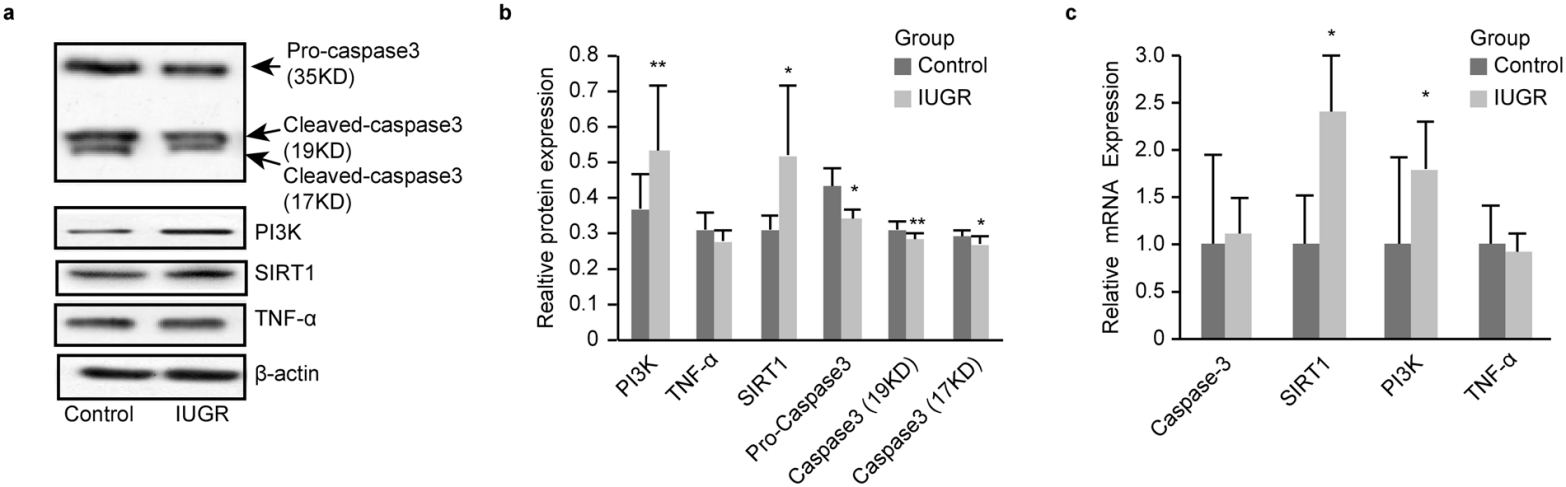
Hippocampal Caspase-3, PI3K, SIRT1 and TNF-α protein and mRNA expression levels on DOL1. (**a**) Representative Western blots for caspase-3, PI3K, SIRT1, TNF-α and β-actin (all groups, n = 8 rats). (full-length blots/gels are presented in Supplementary Fig. S5). (**b**) Representative Western blotting results for PI3K, TNF-α, SIRT1, pro-caspase3, cleaved-Casp3 (19KD) and cleaved-Casp3 (17KD) in control and IUGR groups. 35KD is pro-Casp3, 17KD and 19KD are cleaved Casp3. (**c**) Representative Casp3, SIRT1, PI3K and TNF-α mRNA expression levels as determined via RT-PCR in two groups. The values are expressed as the mean ± SD. *P < 0.05, **P < 0.01.

On DOL1, SIRT1 and PI3K mRNA expression levels were significantly higher in the IUGR group (all *P* < *0*.*05*); however, the Casp3 and TNF-α mRNA expression levels were not significantly different between the two groups [Fig. 3c].

**Figure 4:**
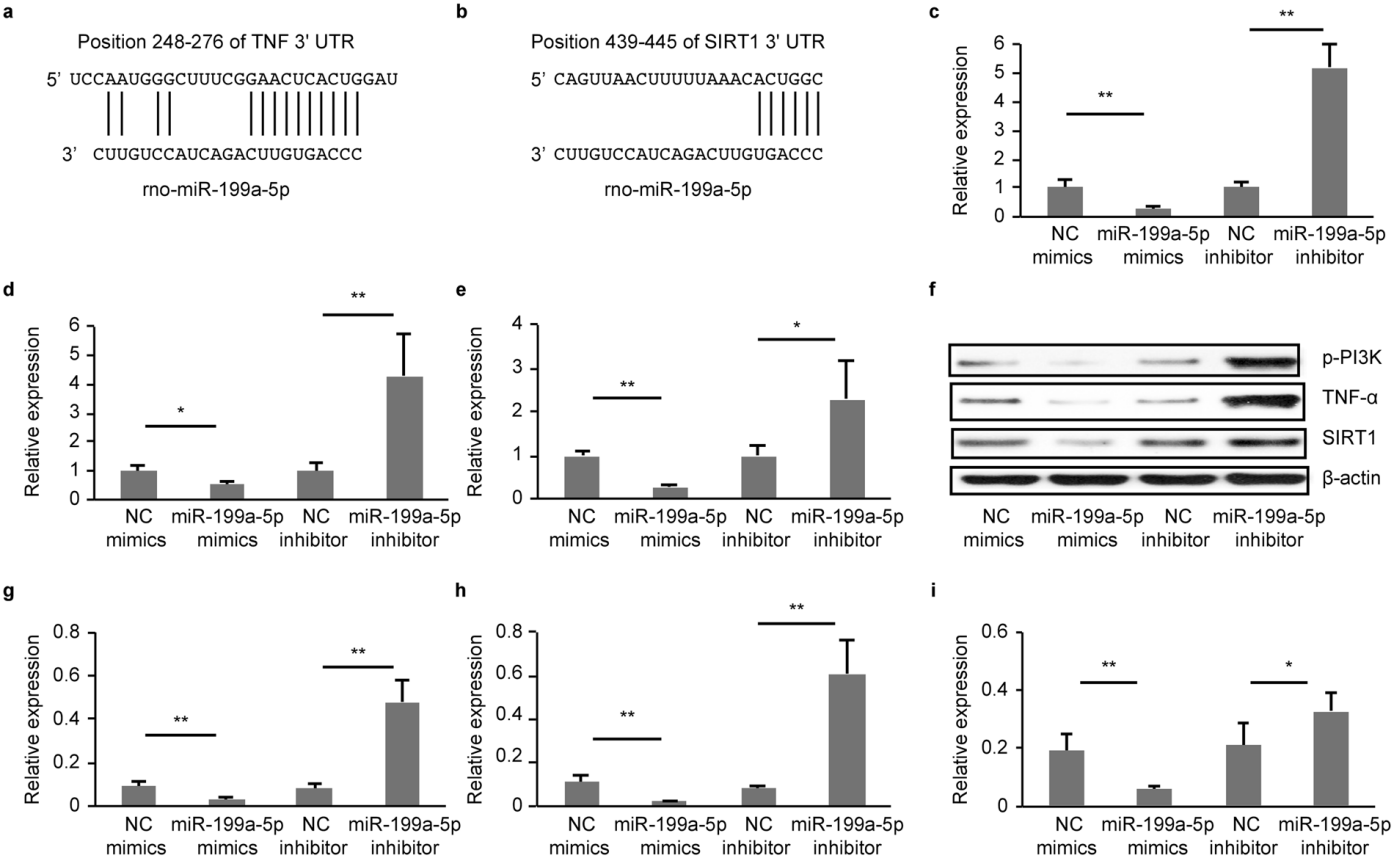
MiR-199a-5p inhibitors PI3K, TNF-α and SIRT1. (**a**,**b**) The alignment between Mus musculus miR-199a-5p and the 3-UTR of TNF-α and SIRT1, identified by TargetScanS software. (**c**–**e**) qRT-PCR analysis of PI3K (**c**), TNF-α (**d**) and SIRT1(**e**) mRNA expression levels in the PC12 cells following transfection. (**g**–**i**) Western blot analysis of PI3K (**g**), TNF-α (**h**) and SIRT1 (**i**) protein expression in PC12 cells following transfection. (full-length blots/gels are presented in Supplementary Fig. S7). The values are expressed as the mean ± SD. *P < 0.05, **P < 0.01.

**Figure 5:**
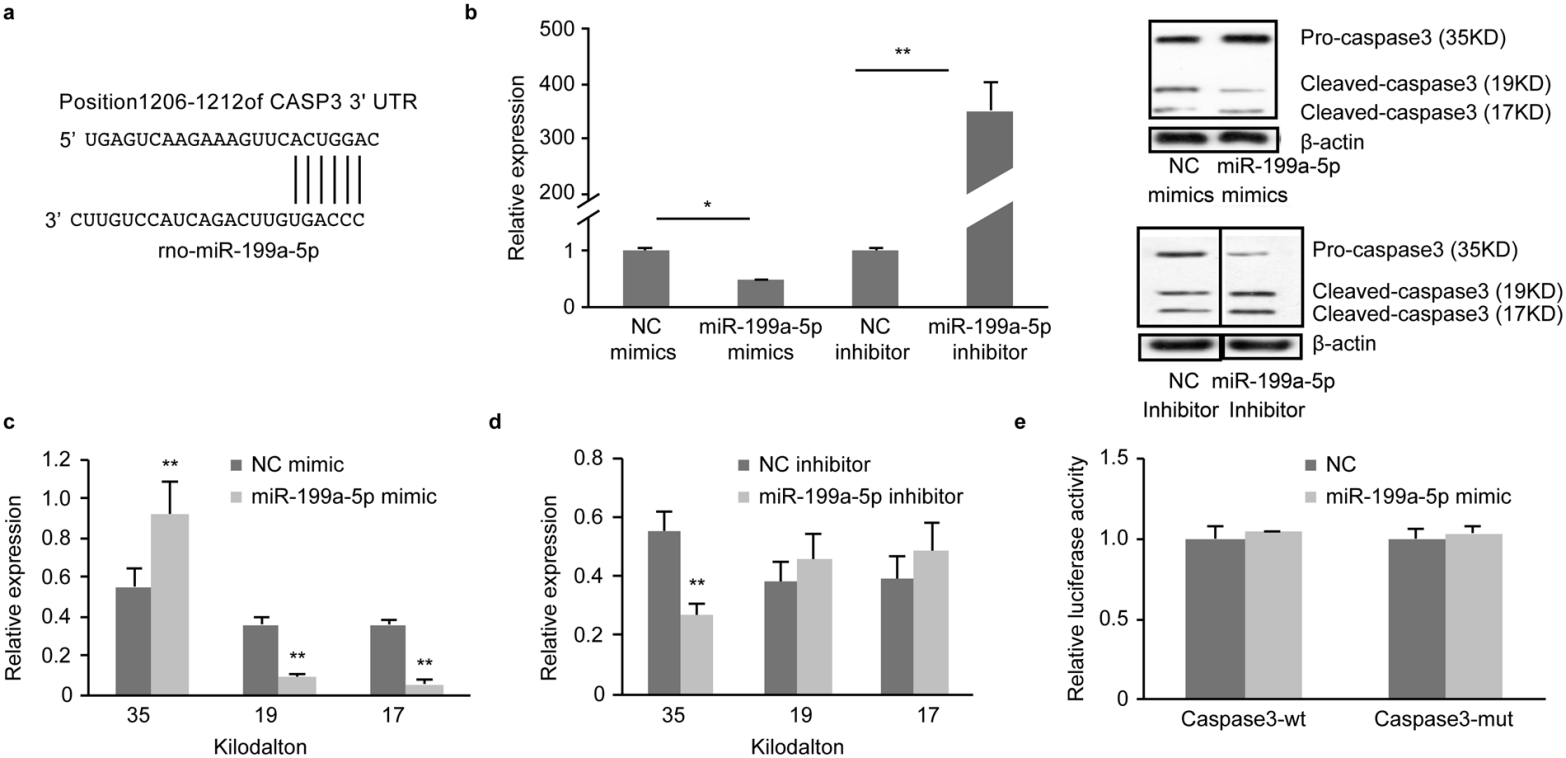
Casp3 isn’t target of miR-199a-5p. (**a**) The alignment between Mus musculus miR-199a-5p and the 3-UTR of Casp3, identified by TargetScanS software. (**b**) qRT-PCR analysis of Casp3 mRNA expression levels in the PC12 cells following transfection. (**c**,**d**) Western blot analysis of Casp3 protein expression in PC12 cells following transfection. (full-length blots/gels are presented in Supplementary Fig. S8). (**e**) Comparison of luciferase activity in the NC and miR-199a-5p mimic groups. The values are expressed as the mean ± SD. *P < 0.05, **P < 0.01.

The Western blots results indicated that IUGR significantly lowered hippocampal Casp3 expression [35-kDa pro-Casp3, 19-kDa and 17-kDa cleaved caspase-3] (*P* < 0.05, 0.01, and 0.05, respectively) and increased SIRT1 and PI3K expression (*P* < 0.05 and 0.01, respectively) [Fig. 3b]. The three prediction algorithms showed that miR-199a-5p could bind to the TNF-α, SIRT1 and caspase-3 mRNA 3′UTRs [Figs 4a and 5a]; therefore, we chose them for the subject of next step.

### MiR-199a-5p inhibited PI3K, TNF-α and SIRT1

Previous studies have demonstrated that miR-199a-5p could bind to 3′UTR of SIRT1 and TNF-α mRNA, and inhibitor PI3K^11–13^. To determine the targets of miR-199a-5p, we transfected miR-199a-5p miRNA mimics into a rat pheochromocytoma-derived cell line (PC12 neuron-like cells) and compared our results with a miRNA mimic negative control. Using RT-PCR, we determined that PI3K, TNF-α and SIRT1mRNA expression was significantly decreased following transfection with miR-199a-5p mimics (*P* < 0.01, 0.05, and 0.01, respectively) [Fig. 4c–e].Western blotting demonstrated that PI3K, TNF-α and SIRT1 protein expression levels in the group treated with the miR-199a-5p mimics were significantly lower than the control group (*P* < 0.01, 0.01, and 0.05, respectively) [Fig. 4g–i]. The expression levels of PI3K, TNF-α and SIRT1 were consistent with above results when miR-199a-5p miRNA mimics was transfected into rat primary neurons [Fig. S4h–j].

To block endogenous miRNA functions, we transfected miR-199a-5p inhibitors into PC12 cells and compared our results with a miRNA inhibitor negative control. Using a real-time quantitative RT-PCR assay, we observed that the introduction of the miR-199a-5p inhibitors increased PI3K, TNF-α and SIRT1 mRNA expression levels (5-, 4- and 2-fold, respectively) [Fig. 4c–e]. Western blotting demonstrated that PI3K, TNF-α and SIRT1 protein expression levels in the group treated with the miR-199a-5p inhibitor were significantly higher than those in the control group (*P* < 0.05, 0.01, and 0.05, respectively) [Fig. 4g–i]. The expression levels of PI3K, TNF-α and SIRT1 were consistent with above results when miR-199a-5p miRNA inhibitor was transfected into rat primary neurons [Fig. S4h–j].

### Casp3 was not a target of miR-199a-5p

To determine the targets of miR-199a-5p, we transfected miR-199a-5p miRNA mimics into a rat pheochromocytoma-derived cell line (PC12 neuron-like cells) and compared our results with those of a miRNA mimic negative control. We determined that Casp3 mRNA expression was significantly decreased following transfection with the miR-199a-5p mimics (*P* < 0.05) [Fig. 5b]. Western blotting demonstrated that cleaved-Casp3 (19 kDa and 17 KDa) expression was significantly decreased than those in the control group (*P* < 0.01 and 0.01, respectively) [Fig. 5c]. Casp3 expression levels were consistent with above results when miR-199a-5p miRNA mimics were transfected into rat primary neurons [Fig. S4k].

To block endogenous miRNA functions, we transfected miR-199a-5p inhibitors into PC12 cells and compared our results with those of a miRNA inhibitor negative control. We observed that the introduction of the miR-199a-5p inhibitor increased Casp3 mRNA expression levels (352-fold) [Fig. 5b]. Western blotting demonstrated that pro-Casp3 (35 kDa) protein expression levels were significantly lower in the inhibitor-treated group than in the control group (*P* < 0.01) [Fig. 5d]. However, we found that the relative luciferase activity in T293cells transfected with wild-type (Casp3-wt) or mutation-type Casp3 (Casp3-mut) constructs was not different between the miR-199a-5p mimic and negative control groups (*P* > 0.05). Casp3 expression levels were consistent with above results when miR-199a-5p miRNA inhibitor was transfected into rat primary neurons [Fig. S4k].

In summary, our results indicated that miR-199a-5p did not bind to the Casp3 mRNA 3′UTR; however, miR-199a-5p regulated the expression of Casp3 by binding to other genes.

### MiR-199a-5p did not mediate PC12 cells apoptosis by regulating Casp3 expression

The terminal deoxynucleotidyl transferase uridine nick end labelling (TUNEL) assay was used to compare the number of apoptotic cells between the two groups. On DOL1, fewer TUNEL-positive cells were found in the hippocampal area CA1 in the IUGR group compared with the control group (control group 9.5 ± 1.0 TUNEL-positive cells/HPF and IUGR group 7.7 ± 1.0 TUNEL-positive cells/HPF), but the difference between the groups was not significant (*P* = 0.058) [Fig. S2].

To determine whether miR-199a-5p triggered neuronal cell apoptosis, PC12 cell apoptosis was detected after transfection by Annexin V/PI staining and analysis by flow cytometry. Consistent with the above mentioned results, the difference between the groups was not significant (NC mimic group vs. miR-199a-5p mimic group or NC inhibitor group vs. miR-199a-5p inhibitor group, *P* > 0.05) [Fig. S5].

## Discussion

The mechanisms underlying neuronal changes in IUGR are unknown. The primary finding of this study was that the expression of specific types of miRNA in the hippocampus differed significantly between normal rats and rats with IUGR. Because miRNAs modulate mRNA function, we hypothesized that miRNAs may play important roles in the regulation of neuronal development in IUGR. We also demonstrated that miR-199a-5p played a role in regulating SIRT1.

Previous evidence suggests that newborns with IUGR have specific neurostructural and neurodevelopmental anomalies^14^. The brain’s morphological characteristics of infant with IUGR contain decreased numbers of neurons and decreased proportional volumes^15–17^. Animal models have shown that both under- and over-nutrition during pregnancy induce stable alterations in physiological and structural phenotypes in offspring^18,19^. Nutrients are the one of the most important epigenetic factors that affect nervous system development during the foetal period^20,21^.

MiR-199a-5p and miR-199a-3p are both processed from the same precursor, miR-199a. A number of studies have shown that miR-204 and miR-199a were enriched in the brain during early development and played an important role in brain injury^22,23^. After birth, rats with IUGR exhibit catch-up growth; during the catch-up growth period, the development of the brain in rats with IUGR is accelerated (Fig. S1).The epigenetic characteristics (on DOL1 and DOL 7) in the brain in IUGR rats were different from those of the control group; this change may be associated with the changed nutritional status.

Previous studies on rats with IUGR demonstrated that the levels of neuronal apoptosis secondary to uteroplacental insufficiency were increased following a perinatal insult^24–26^. IUGR increased hippocampal apoptosis in IUGR rats, and Casp3 activation was one of the final steps in the execution of apoptosis, with increased Casp3 levels as the most direct sign of increased neuronal apoptosis^27^. Our experimental model was different from the above mentioned studies, and our results are contrary. The programmed death (apoptosis) of neural precursor cells during earlier stages of neural development affects the proliferation of neural precursor cells and young postmitotic neuroblasts, and a low-protein diet can decrease neuronal apoptosis according to the neurotrophic theory^27,28^. Previous studies have shown that brain volume was reduced following a reduction in the number of nerve cells^3,29^. Previous studies have also demonstrated that Casp3 deficiency decreased the number of progenitor cells lost to programmed cell death, resulting in marked dysplasia and nervous system malformations^30^. Therefore, we believe that decreased Casp3 expression may be important in the pathogenesis of neurological morbidities in IUGR caused by malnutrition.

Previous studies have confirmed the relationship between miR-199a and apoptosis^31–34^, and bioinformatics analysis has shown that Casp3, a key initiator of apoptosis, was a hypothetical target of miR-199a-5p. Our results indicated that miR-199a-5p did not bind to the Casp3 mRNA 3′UTR; however, miR-199a-5p regulated the expression of Casp3 by binding to other genes. Moreover, flow cytometry results were also negative. We believe that the following two hypotheses can account for these results: (1) the HiPerFect reagent is cytotoxic, possibly affecting the apoptosis of PC12 cells and (2) miR-199a-5p regulated the expression of Casp3 by binding to other genes, but this had little effect on neuronal apoptosis.

SIRT1 is found in the hippocampus, striatum, cerebral cortex and cerebellum and is involved in regulating neuronal differentiation and apoptosis, learning and memory formation^35^. Increased SIRT1 expression prior to IUGR has been confirmed in previous studies, and SIRT1 is an important protective factor against IUGR^36,37^. In our study, SIRT1 expression in the hippocampus was also increased. SIRT1 presents as a response to low nutritional availability in IUGR pathogenesis, with increased SIRT1 levels precipitating increased lipogenesis because it decreases Casp3 activity^38,39^. SIRT1 is a predicted target of miR-199a, which has been confirmed in other models^40^. Thus, our results were consistent with those of previous experiments. Previous studies have demonstrated that the PI3K/Akt signalling pathway and TNF-α played vital roles in neural development^41,42^. Some studies also have shown that miR-199a-5p can regulate PI3K and TNF-α. Consistently, our study also showed that miR-199a-5p decreased the expression of PI3K and TNF-α^12,13^.

## Conclusion

We demonstrated that the expression levels of specific miRNAs in the hippocampus differed significantly between normal and IUGR rats, and these miRNAs may play important roles in the regulation of neuronal development. Additionally, we demonstrated that miR-199a-5p suppressed SIRT1 and PI3K and regulated the expression of Casp3 but could not directly bind Casp3, all of which affect hippocampal development.

## Materials and Methods

### Animals and treatments

All experiments were executed in accordance with the Guide for the Care and Use at Laboratory Animals and were approved by Research and Ethics Committee at Central South University of Medical Sciences. We confirmed successful mating (Sprague-Dawley rats; 18 females, 9 males; 3 months of age) using vaginal spears, and evidence of sperm on the vaginal spear was considered day zero of gestation. Pregnant dams were singly housed and subjected to either a normal diet with a protein content of 21% (w/w) or a low-protein diet with a protein content of 10% (w/w). The Day 1 pups (day of life 1; DOL1) were delivered at term, and experimental samples were harvested on DOL 1, 7, 21 (3 weeks), and 56 (8 weeks). The brains were quickly removed and either dissected to isolate the hippocampus before flash-freezing for hippocampus collection or processed for histological evaluation.

### MiRNA microarray

The miRNA microarray analysis was performed at Shanghai Biotechnology Corporation (Shanghai, China) according to a procedure that was described in detail at the website of the corporation (http://www.ebioservice.com). Briefly, 5 μg of RNA from each DOL7 hippocampus tissue was isolated by using the mirVana RNA Isolation Kit (Applied Biosystems p/n AM1556, Applied Biosystems, Foster City, CA, USA). Following 3/-extension with a poly(A) tail using poly(A) polymerase, 3DNA dendrimers were ligated to the total RNA to allow multiple biotins (~15) to bind to the poly(A)-tail of each RNA molecule. After flash tag ligation (Affymetrix, Santa Clara, CA, USA), hybridization was performed on a miRNA microarray chip (Affymetrix miRNA 3.0). The hybridization signals were detected using GeneChip Scanner 7 G (Affymetrix). The raw data were normalized and analysed using the software Command Console 3.2 (Affymetrix). Data analysis was carried out by the method of quantitative log2 metrics. A 2-fold change in expression was chosen as the cut-off to determine the significance of between-group differences.

### Quantitative real-time polymerase chain reaction (qRT-PCR)

The TRIzol reagent (Invitrogen, USA) was used to isolate total RNA from the hippocampus on DOL1 and DOL7. miRNAs were polyadenylated by poly(A) polymerase and reverse transcribed to cDNA using the One Step PrimeScript^®^ miRNA cDNA Synthesis Kit (TaKaRa, China). Real-time quantitative PCR was performed with the SYBR^®^ Premix Ex Taq™ II kit (TaKaRa, China). All protocols were implemented according to the manufacturer’s instructions. The U6 nuclear RNA was used as the reference control.

The expression levels of the target genes TNF-α, SIRT1, PI3Kand Casp3 were also quantified using qRT-PCR (SYBR^®^ Premix Ex Taq™ II kit). The reverse transcription of Casp3, SIRT1 and TNF-α was performed using the PrimeScriptTM RT reagent kit with gDNA Eraser (Takara Bio Inc.). β-actin was used as an internal control for mRNA RT-qPCR analysis. All genes were amplified in separate wells in triplicate. The fold change was calculated using the 2^−ΔΔCt^ method. The primers sequences are listed in Table 2.

**Table 2:** The primer sequences for RT-qPCR.

Gene	Primer Sequence
SIRT-1	F:5′-GGAACCTCTGCCTCATCTAC-3′
	R:5′-GCATACTCGCCACCTAACC-3′
PI3K	F:5′-GATGAGGTGAGGAACGGAAGAATG-3′
	R:5′-CGGTCACAGTCCCACAAAGA-3′
Caspase-3	F:5′-GCTTGTCGGCATACTGTTTCAG-3′
	R:5′-AGAACTGGACTGTGGCATTGAG-3′
TNF-α	F:5′-CGTGTTCATCCGTTCTCTA-3′
	R:5′-ATCTTCAGCAGCCTTGTG-3′
β-actin	F:5′-AGG CCCCTCTGAACCCTAAG-3′
	R:5′-CCAGAGGCATACAGGGACAAC-3′
miR-199a-5p	F:5′-AGTGTTCAGACTACCTGTTC-3′
miR-199a-3p	F:5′-ACAGTAGTCTGCACATTGGTTA-3′
miR-204	F:5′-TTCCCTTTGTCATCCTATGCC-3′
miR-34C	F:5′-GCCGTGTGTTAGTGATTG-3′
miR-219	F:5′-GATGTCCAACGCAATTCT-3′
U6	F:5′-TGGCCCCTGCGCAAGGATG -3′

### Target prediction and Kyoto Encyclopaedia of Genes and Genomes pathway analyses

Three bioinformatics algorithms were used to predict the target genes of the differentially expressed miRNAs in the hippocampi of rats with IUGR, including miRanda (http://microrna.sanger.ac.uk/targets/v4.0), MirWalk (http://www.umm.uni-heidelberg.de/apps/zmf/mirwalk/), and TargetScan (http://www.targetscan.org/). KEGG pathway analyses were performed using the DAVID bioinformatics resources (http://david.abcc.ncifcrf.gov/).

### Protein isolation and Western blotting

Western blots were performed using rabbit anti-SIRT1 (Santa Cruz, CA, USA), anti-PI3K(Abcam), anti-Casp3 (CST, MA, USA), and anti-TNF-α (CST) antibodies as well as a β-actin antibody (Abcam). The hippocampi were homogenized in ice-cold lysis buffer, and the protein concentrations were determined using the bicinchoninic acid (BCA) method (Pierce, Rockford, IL) following centrifugation. The proteins were separated using 10% sodium dodecyl sulphate–polyacrylamide gel electrophoresis (SDS-PAGE)-ready gels (Bio-Rad, Hercules, CA) and then electro-transferred onto nitrocellulose membranes. After blocking the membranes with 5% milk in Tris-buffered saline (TBS) for 1 h, we exposed the bound proteins to antibodies overnight at 4 °C. Following extensive washing with TBST, a 1:2,000 dilution of goat anti-rabbit horseradish peroxidase (HRP) secondary antibody (Cell Signaling Technology) was applied, and the membrane was incubated for 1 h at room temperature. Following extensive washing with TBST, the blots were examined using Western Lightning ECL (Perkin Elmer Life Sciences), with β-actin as a normalization control. The protein band intensities were determined by densitometric analysis using the Image J software.

### Cell culture and treatments

PC12 cells were cultured in Dulbecco’s Modified Eagle Medium (DMEM) supplemented with 10% foetal bovine serum, antibiotics, and 1% L-glutamine, The cells were then treated with 100 ng/ml nerve growth factor (NGF, Sigma) [Fig. S3]. The cells were maintained at 37 °C in a humidified 5% CO2 incubator, and the medium was refreshed every 2–3 days while the cells differentiated into a neuron-like phenotype.

### Primary neuronal culture and immunofluorescence assay

Primary hippocampal neurons were cultured from 18 neonatal rat brains and incubated in neurobasal media supplemented with B-27, 0.5 mM L-glutamine, 12.5 μM glutamate, 100 units/ml penicillin and streptomycin (all from Invitrogen, USA) on tissue culture plates coated with poly-D-lysine (Sigma, USA). The neurons were maintained by changing medium (without glutamate) every 2 ± 3 days. All experiments were performed at 7 ± 14 DIV. 5 μg/ml of α-syn aggregates were added to a 12-well plate and 1 μg/ml to a 96-well plate. Neurons grown on culture slides were fixed, blocked with 5% donkey serum at room temperature for 1 h, incubated with primary antibody (anti-rat neuron-specific enolase (NSE), 1:100, Abcam) at 4 °C overnight, and finally incubated with secondary antibody (anti-mouse IgG) at room temperature for 1 h. Positive staining was visualized using a confocal fluorescence microscope.

### Transfection

One day before transfection, the cells were seeded into 6-well tissue culture plates at a density of 5–8 × 10^4^ cells per well for RNA preparation 48–72 h post-transfection. PC12 cells and primary neuron transfection was accomplished using the HiPerFect transfection reagent (Qiagen). Briefly, we divided the cells into blank, negative control (NC), miR-199a-5p mimic, NC inhibitor (5′-CAGUACUUUUGUGUAGUACAA-3′), and miR-199a-5p inhibitor (5′-GAACAGGUAGUCUGAACACUGGG-3′) groups. The cells were washed once with Opti-MEM (Invitrogen) before 500 μl Opti-MEM was added to each well. 100 nM miRNA duplexes (GenePharma, China) and mimic or inhibitor (100 pmol, GenePharma) were mixed in 250 μl Opti-MEM; 250 μl Opti-MEM containing 7.5 μl HiPerFect was mixed with the above mixture and then incubated for 20 min at room temperature. The combined mixture was then added to the cells in a 6-well plate for a transfection volume of 1 ml. The Opti-MEM medium containing the complexes was replaced with 2 ml standard growth medium. Samples were collected following transfection and stored at −80 °C for subsequent analysis. Real-time reverse transcription-PCR analysis of miR-199a-5p, PI3K, TNF-α, SIRT1 and Casp3 mRNA and Western blot analyses for the protein expression levels of PI3K, TNF-α, Sirt1 and caspase-3 were performed. The tests were repeated in triplicate.

### Dual-luciferase reporter assay

The 3′UTR sequences of caspase3 and its corresponding mutant (mt) 3′UTR sequences were cloned into the pmiR-RB-Report™ vector (RIBOBIO, Guangzhou, China). Then, 293 T cells were added into a 24-well plate and transfected with pmiR-Casp3-wt or pmiR-caspase-mut and 100 nM miR-199a-5p mimics or NC with lipofectamine 2000. Cells were analysed using the Dual-Luciferase Reporter Assay System (Promega) after 48 h according to the manufacturer’s protocol using a Tecan M1000. The experiment was repeated three times, each time in triplicate.

### TUNEL staining

Apoptotic nuclei were labelled using the terminal deoxynucleotidyl transferase uridine nick end labelling (TUNEL) technique with an *In Situ* Death Detection Kit, POD (Roche, Nutley, NJ, USA). A light haematoxylin stain was used for tissue orientation following 3, 3′-diaminobenzidine (Sigma)/H_2_O_2_ rinses. The number of apoptotic cells stained by TUNEL assay in the hippocampal CA1 region was counted as following: An observer blinded to the study group counted the total number of apoptotic nuclei per high-power field (HPF) in six non-overlapping regions per section (×400).

### Annexin V/propidium iodide apoptosis assay

PC12 cells treated with stigma asterol were incubated for 24 h in 6 cm^2^ dishes at a cell density of 1 × 10^6^ cells/ml. After 24 h, the cells were washed 2 times, detached and then incubated either in the presence or absence of different chemical reagents. These cells were washed twice with ice-cold PBS, and resuspended with binding buffer, then added into Annexin V-FITC for 15 min and added to binding buffer containing propidium iodide (PI) for 5 min at room temperature in the dark. The samples were then analysed by flow cytometry (FACS Calibur, BD Sciences, Heidelberg, NJ, USA) within 1 h.

### Statistical analysis

The values included in the text and figures are expressed as the mean ± standard deviation (SD). Comparisons between groups were performed using Student’s t-test. N indicates the number of rats, and a *P*-value < 0.05 was considered statistically significant.

## Electronic supplementary material

Supplementary Information

## Data Availability

All data generated or analysed during this study are included in this published article (and its Supplementary Information files). The datasets generated during and/or analysed during the current study are available from the corresponding author on reasonable request.
