# Supplementary material for: MiR-199a-5p regulates sirtuin1 and PI3K in the rat hippocampus with intrauterine growth restriction
Source: Sci Rep. 2018 Sep 14;8:13813. doi: 10.1038/s41598-018-32189-5 (PMC6138635; doi:10.1038/s41598-018-32189-5)
Supplement: Supplementary file 1 — Supplementary Information [file 41598_2018_32189_MOESM1_ESM.pdf]

MiR-199a-5p regulates sirtuin1 and PI3K in the rat hippocampus with intrauterine growth restriction

Juncao Chen [1, 2], Xiaoyun Gong [2], Li Huang[2], Pingyang Chen [2*], Tao Wang,Wei Zhou[3], Kaiju Luo[2],Jing Wang[2]

1, Institute of Pediatrics, Guangzhou Women and Children's Medical Centre,

Guangzhou Medical University, Guangzhou 510623, China

2, Division of Neonatology, The Second Xiangya Hospital, Central South University, 139 Renmin Middle Rd, Changsha, Hunan, China, 410011.

3. Division of Neonatology, Guangzhou Women and Children's Medical Centre,

Guangzhou Medical University, Guangzhou 510623, China

Corresponding author: Pingyang Chen. Email: chenpingyang@CSU.edu.cn

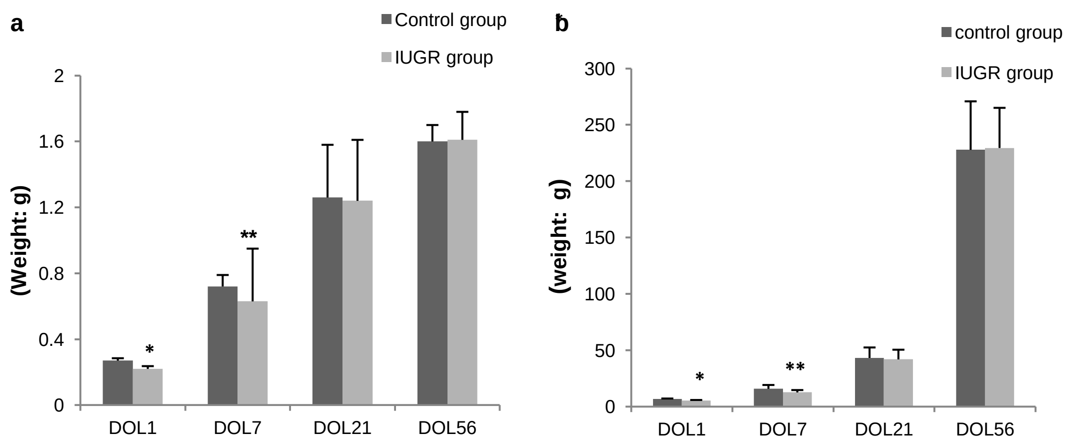

1. **Fig. S1. Brain weights and Body weight at different times.** (**a**) The weight of brain between the control and IUGR groups at different times after birth. (**b**) The body weight between the control and IUGR groups at different times after birth. The values are expressed as the mean±SD. *P<0.05, **P<0.01.

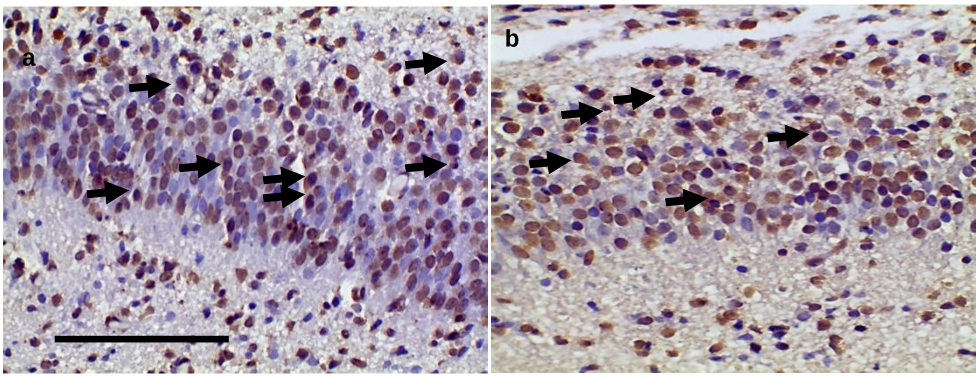

2. **Fig. S2. Apoptotic nuclei stained using the TUNEL technique in the hippocampi CA1 region of the control group (a) and the IUGR group (b) of the DOL1 rats.** Original magnification ×400. Scale bars = 200μm.

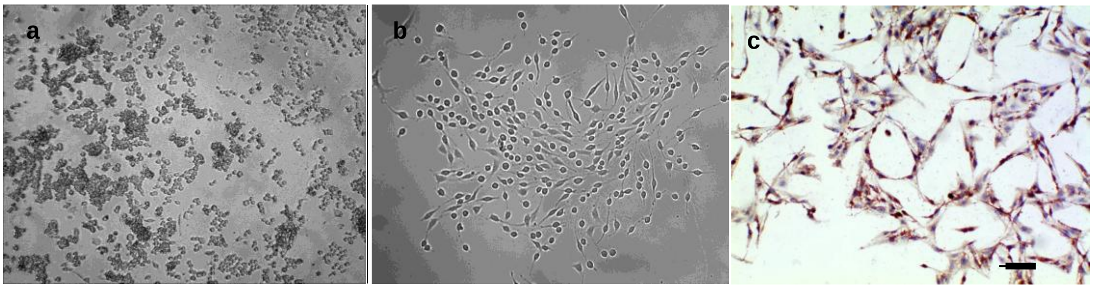

3**. Figure S3. PC12 Cell culture and treatment. (a)** PC12 cell culture. **(b)** PC12 cells treated by NGF. **(c)** PC12 cells staining by NSE. Original magnification ×100.

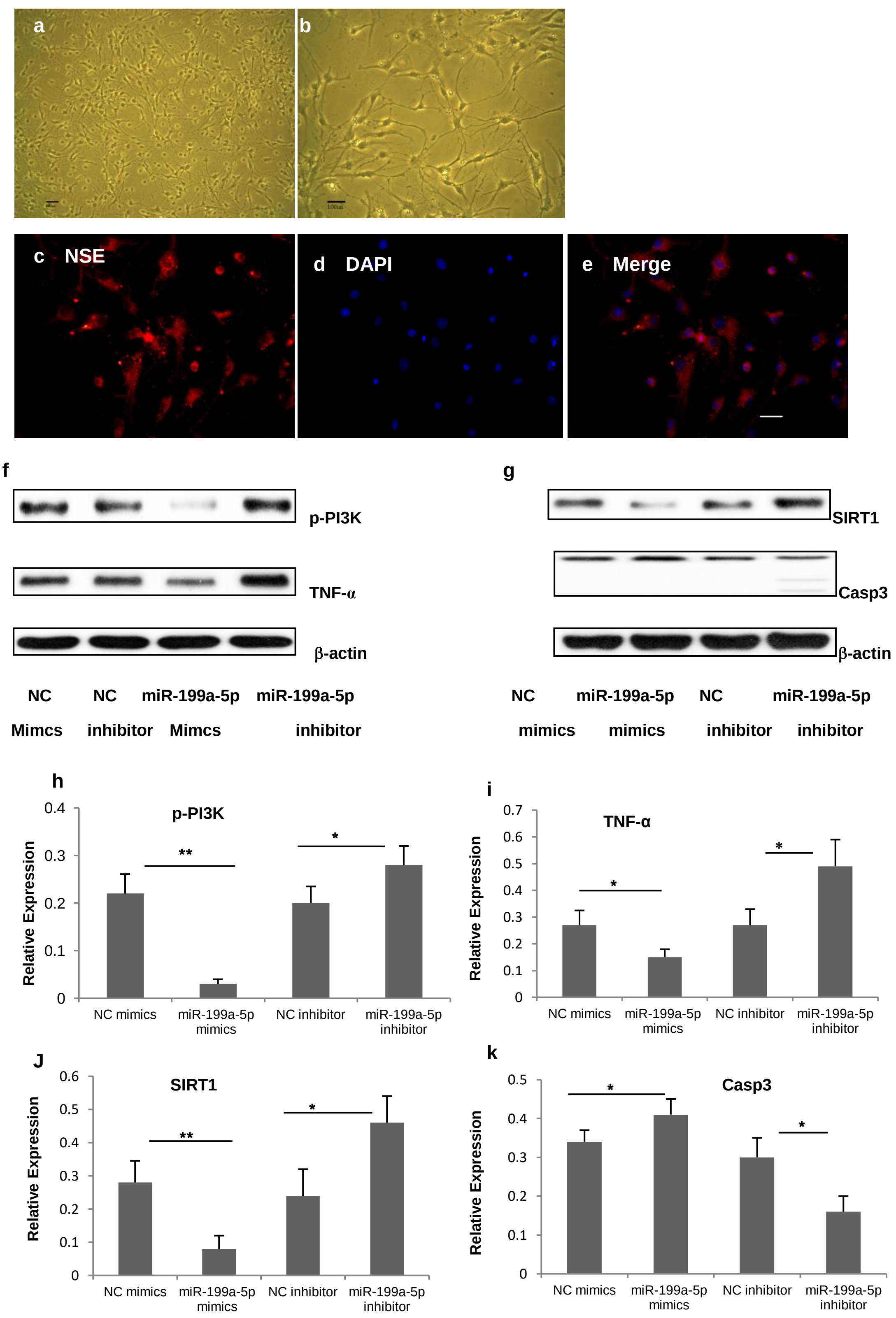

4. **Fig. S4. MiR-199a-5p inhibitors PI3k,TNF-α, SIRT1 and Casp3.** (**a, b**). primary neuronal culture.a, ×100;b, ×200. （**c, d, e**）primary neurons staining by NSE and DAPI. **(h, i, j, k**) Western blot analysis of p-PI3K,TNF-α, SIRT1 and Casp3 protein expression in primary neurons following transfection. Scale bars = 100μm.

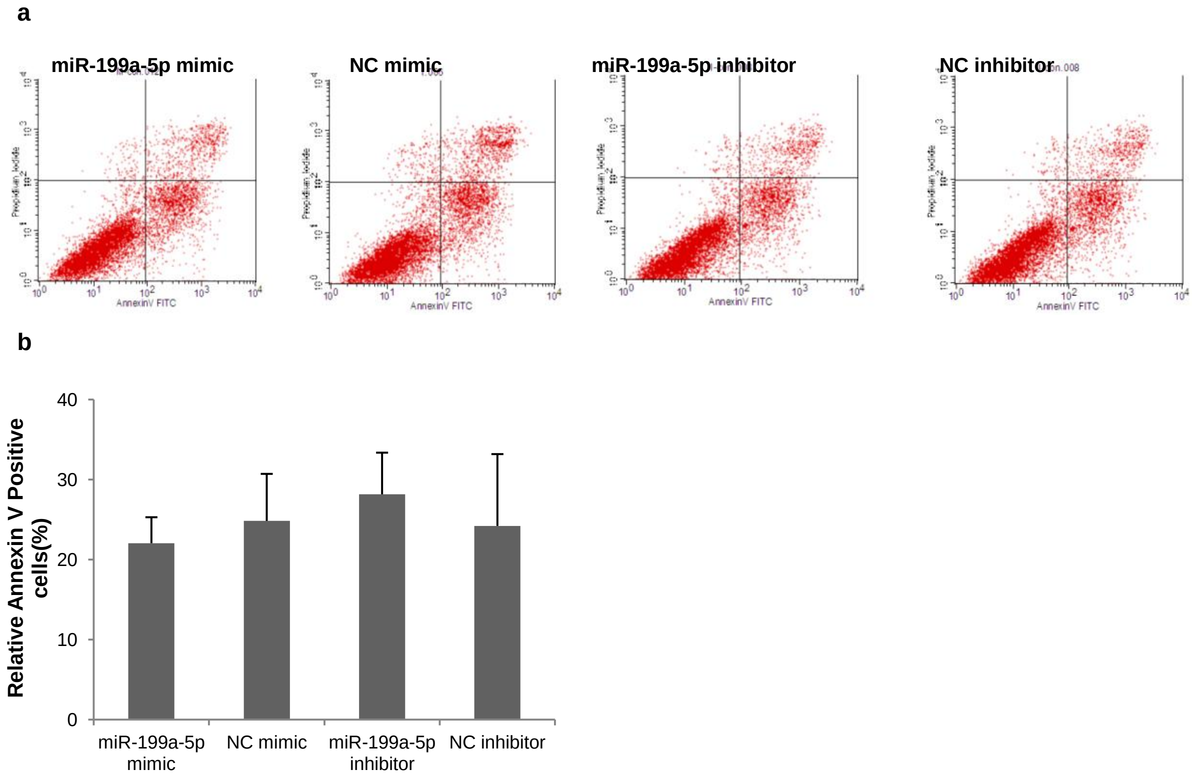

5. **Fig. S5. Effect of miR-199a-5p on PC12 cells apoptosis detected by flow cytometer. (a)** Flow cytometric analysis of PC12 cells were treated with miR-199a-5p mimics and inhibitors. **(b)** Data are presented as the percentage of Annexin Vpositive cells which analyzed by flow cytometer. The values are expressed as the mean ±SD. *P<0.05, **P<0.01.

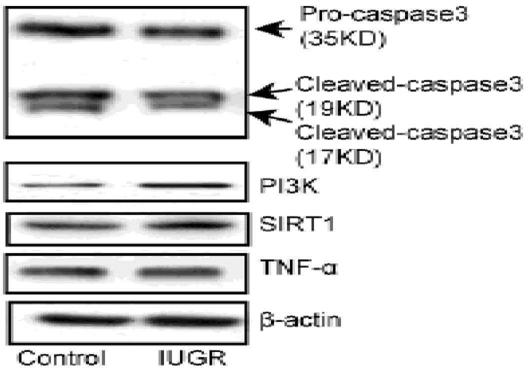

Caspase-3:

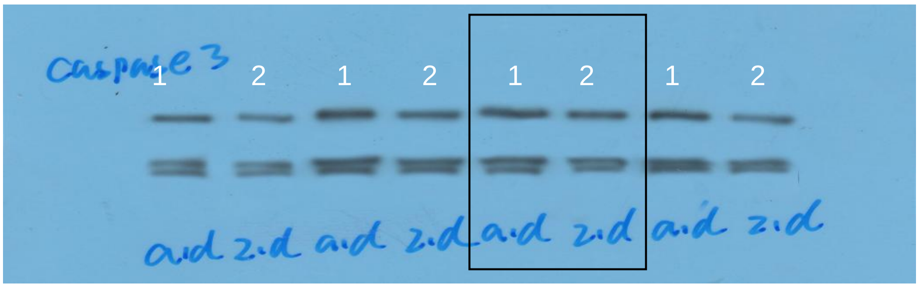

PI3K:

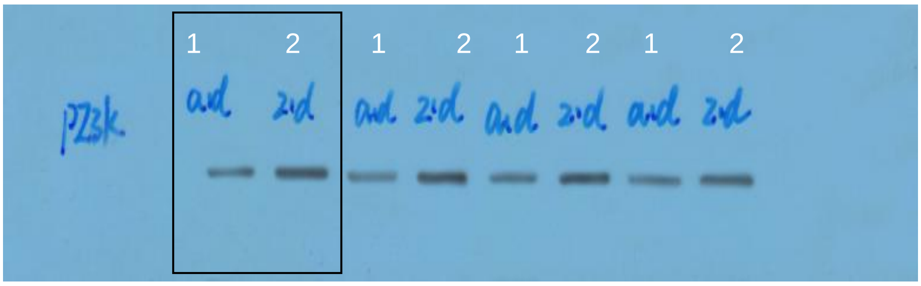

SIRT1:

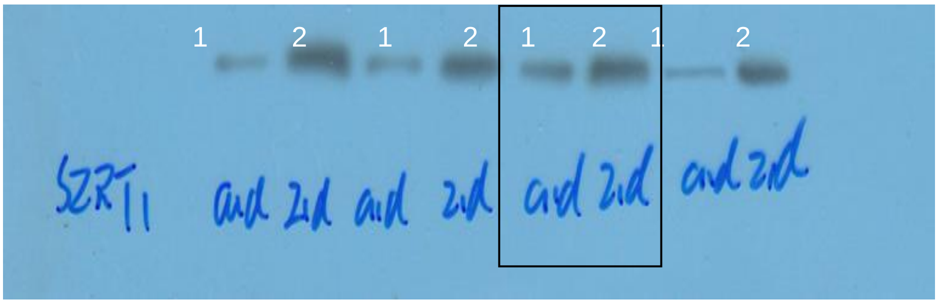

TNF-α:

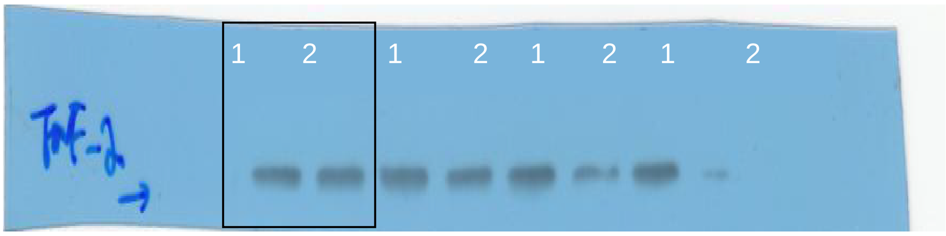

β-actin:

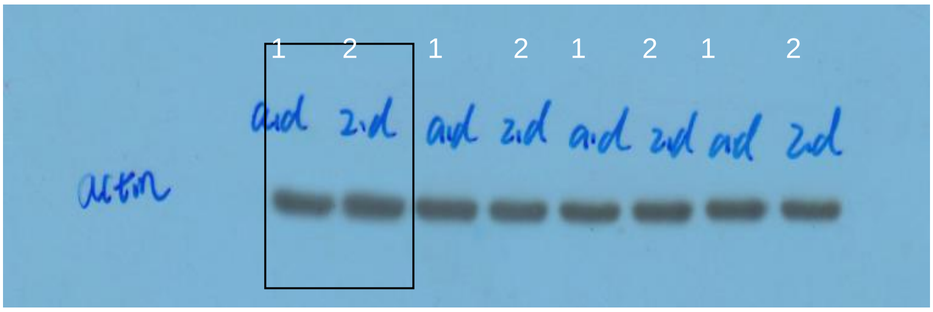

Figure S6. Orignial Western image for Figure 3a. 1, control group; 2, IUGR group.

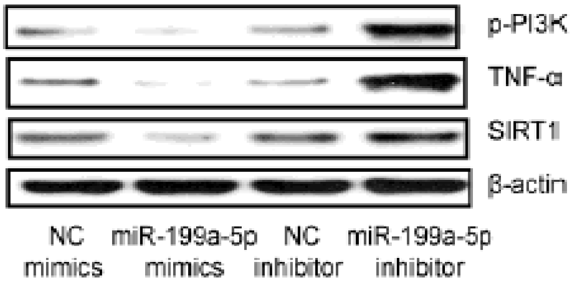

p-PI3K

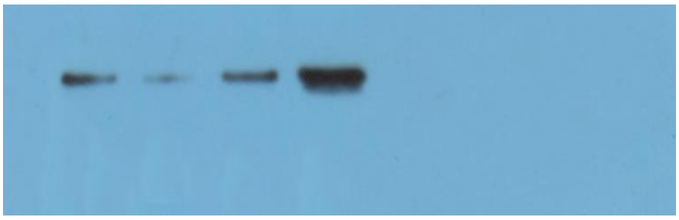

TNF-a

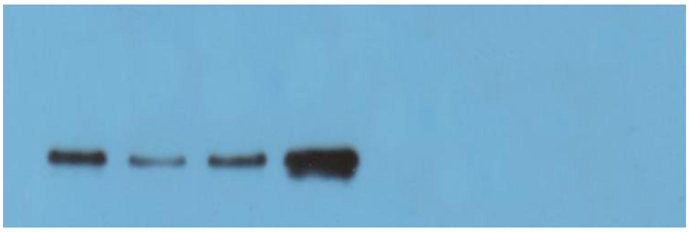

SIRT1

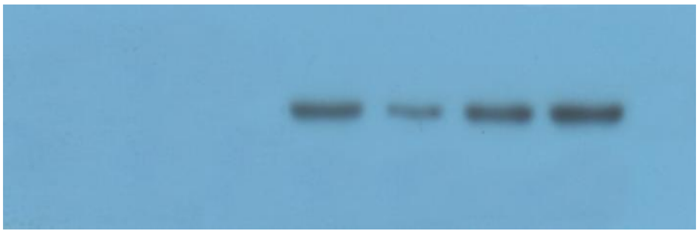

β-actin

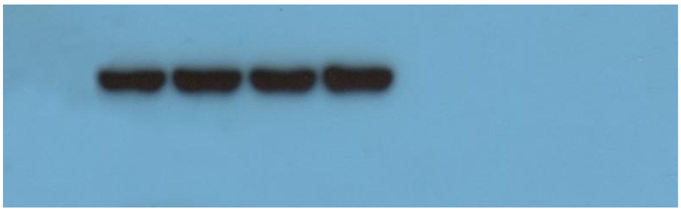

Figure S7. Orignial Western image for Fig. 4f.

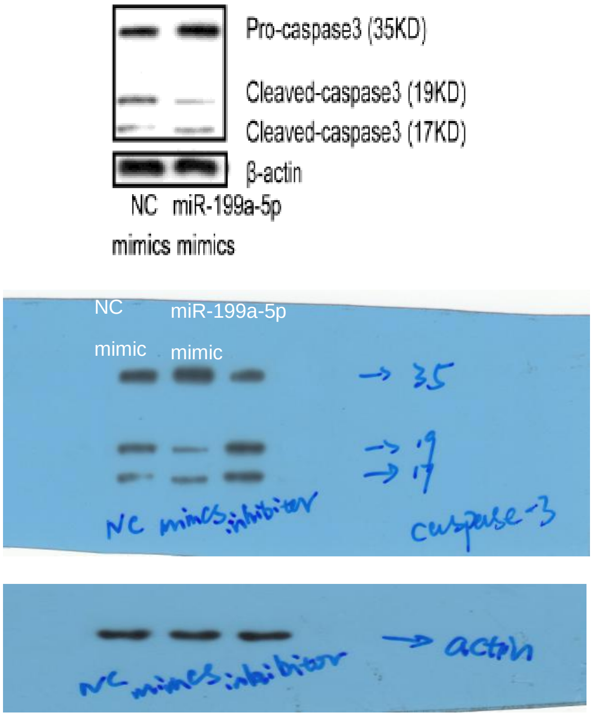

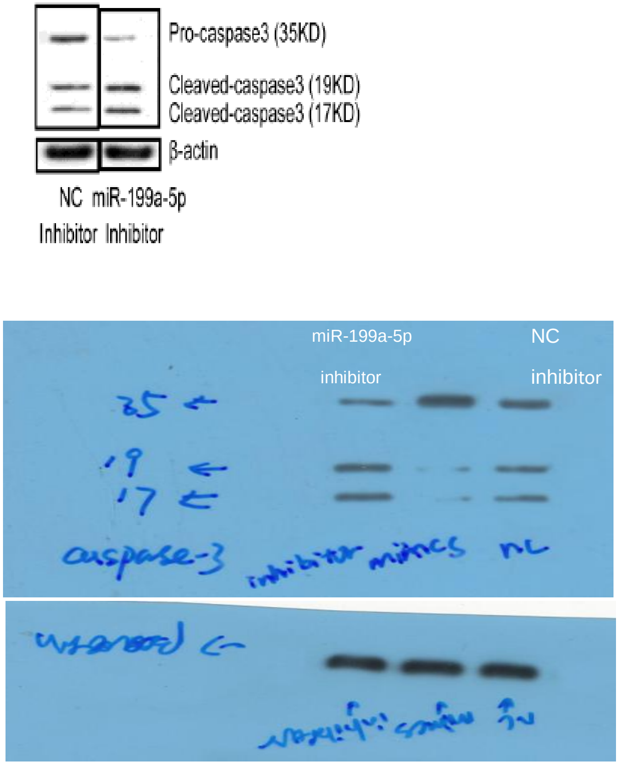

Figure S8. Orignial Western image for Fig. 5b.

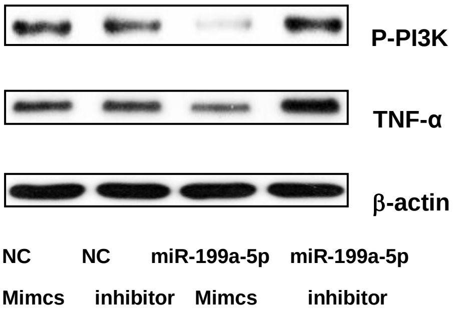

p-PI3K

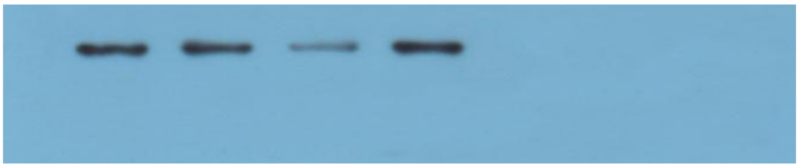

TNF-α

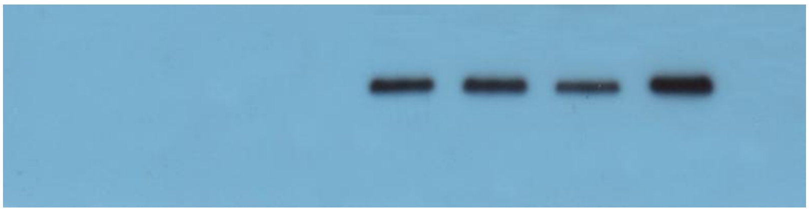

β-actin

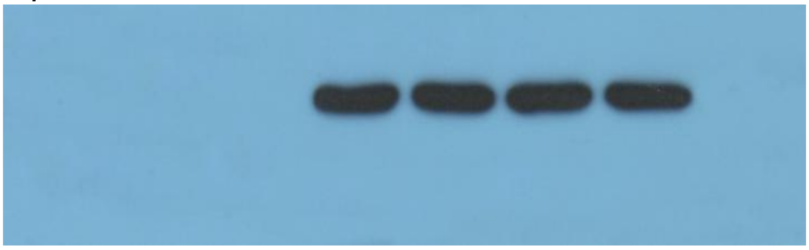

Figure S9. Orignial Western image for Fig. S4f.

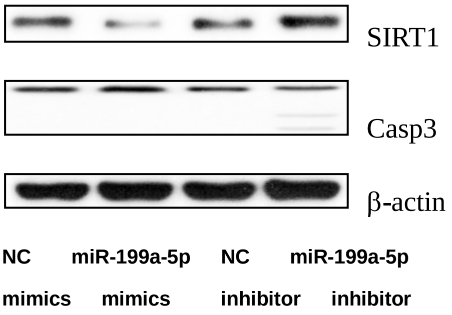

SIRT1

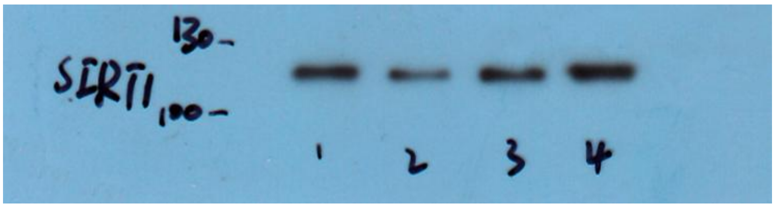

Casp3

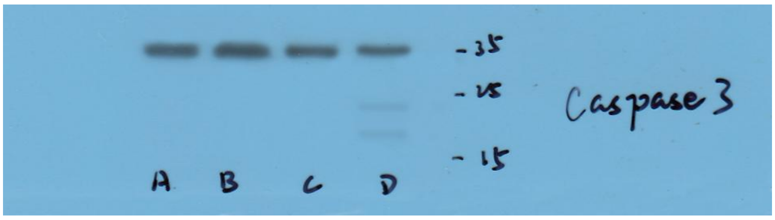

β-actin

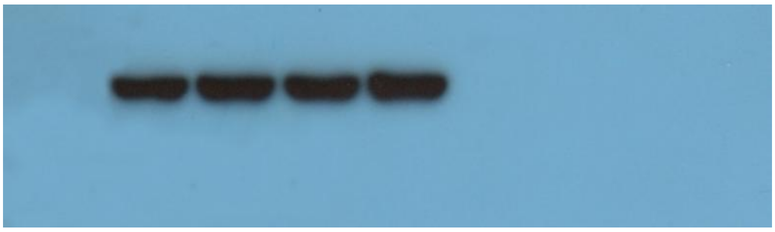

Figure S10. Orignial Western image for Fig.S4g.

Mature miRNA name	Mature sequences (5'-3')		Predicted target gene
rno-miR-199a	rno-miR-199a-5p	CCCAGUGUUCAGACUACCUGUUC	Sirt1,Atp2,Tnf,casp3, pole3,acer2,Sumo3,pi3k
	rno-miR-199a-3p	ACAGUAGUCUGCACAUUGGUUA	Rab6a,Bre,Aspa,Ankfy1, sgsm2,Depdc5,March10
rno-miR-325	rno-miR-325-5p	CCUAGUAGGUGCUCAGUAAGUGU	Trip11,Pla2g2c,PIk1,Zfp407, Ncbp3,Tnfsf9,Nr2f2
	rno-miR-325-3p	UUUAUUGAGCACCUCCUAUCAA	Npc2,vamp7,Fpr2,Prmt1, IkIf1,Nol8,cdn1
rno-mir-219	rno-miR-219a-1-3p	AGAGUUGCGUCUGGACGUCCCG	Bcor,Styl5,Tspan7,Lancl3,cybb,Dynlt3,Rbm3
	rno-miR-219a-5p	UGAUUGUCCAAACGCAAUUCU	Otc,Hdac6,Apex2,Rrag13, Pdha1,Pola1,Flt3
rno-miR-664	rno-miR-664-1-5p	CUGGCUGGGGAAAAAGAUUGG	Slc25a44, Dicer1, Zbtb34, Ncoa1,Insm2,Dnai2,Slco1b2
	rno-miR-664-3p	UAUUCAUUUACUCCCCAGCCUA	Paip1, Ndrg4, Ndrg4, Hey2, Nipal1, Aqp1, Slc2a5, Itga9,
rno-miR-551b	rno-miR-551b-5p	GAAAUCAAGCUUGGGUGAGACCU	Nbl1 ,Casp9, Dctn4, Coq4, Klf12, Nhsl1, Usf1, Hoxa2
	rno-miR-551b-3p	GGCGACCCAUACUUGGUUUCAGU	Srsf5, Btbd7, Ipo7, Skap2, Trak1, Cbll1, Ptprh,Skp2
rno-mir-34C	rno-miR-34c-3p	AAUCACUAACCACACAGCCAGG	Cpsf4, Glrx, Lox, Hdhd2, Polr2c, Rnf2,Lix1, Eepd1
	rno-miR-34c-5p	AGGCAGUGUAGUUAGCUGAUUGC	Bcor,Tspan7,Cybb, Slc38a5, Porcn, Wdr13, Eras, Plp2
rno-miR-195	rno-miR-195-5p	UAGCAGCACAGAAAUAUUGGC	Flot1, Eya3, swt1,Rff1 , Cngn3, Ifitm1, Nodal, Thoc2
	rno-miR-195-3p	CCAAUAUUGGCUGUGCUGCUCCA	Tbp,P2rx4, Szrd1, Lmo1,Cdh8,Sf1,Megf11
rno-miR-204	rno-miR-204-5p	UUCCCUUUGUCAUCCUAUGCCU	Casp3,Sirt1,Zfx,Prima1,Tcea3,Prrc2b,PdcI,Rnd3,Sf1
	rno-miR-204-3p	GCUGGGAAGGCAAAGGGACGUU	Pdcl3,Pla2r1,Banf1,Dtd2, Xpo7,Lrfn2,Lmf1,Timp2
rno-miR-483	rno-miR-483-5p	AAGACGGGAGAAGAGAAGGGAG	Bcor,Otc,Xk,Cybb,Lancl3,Porch,Ebp,Wdr13,Rbm3
	rno-miR-483-3p	CACUCCUCCCCUCCCGUCUUGU	Sra1,Fech,Galr1,Bap1,Scn1b,Git1,Hipk2,Wipf3,Tns3
rno-miR-322	rno-miR-322-5p	CAGCAGCAAUUCAUGUUUUGGA	Lrp6,Ovol1,G2e3,Hoxa4, Lamc2,mitf,Cxcl12,Ret
	rno-miR-322-3p	AAACAUGAAGCGCUGCAACA	Wdr26,Klf8,Gnrhr,Polh,Irf5, Nfib,Bdp1,Gpm6a

rno-miR-329	rno-miR-329-5p	AGAGGUUUUCUGGGUCUCUGUUUC	Nadk2,Ccdc2,Strd4,Gfra3, Cd14,Gde1,Scai,Ehd1
	rno-miR-329-3p	AACACACCCAGCUAACCUUUUU	Brat1,Sox6,Wdtc1,Pbx3, Rgs8,Tead2,Ankfy1,Etv3
rno-miR-196b	rno-miR-196b-5p	UAGGUAGUUUCCUGUUGUUGGG	Xylt1,Tmx3,Ptcd1,neurl1, Nxt2,Cu14b,Wtap,Fv1
	rno-miR-196b-3p	UCGACAGCACGACACUGCCUUCA	Girx5,CIdn2,Zmiz1,Megf11, Gfy,KdeIr1,Smarcc1,Mpp2
rno-miR-449a	rno-miR-449a-5p	UGGCAGUGUAUUGUUAGCUGGU	Calr4,Pan3,Mafk,Gpc2,, Traf3,Rftn2,Sumo1,Naif1
	rno-miR-449a-3p	CAGCUAACAUGCAACUGCUCUC	Nfib,Tnp1,Ikzf2,Sstr1, Smoc2,Mill1,Casp2,Ube2n
rno-miR-324	rno-miR-324-5p	CGCAUCCCCUAGGGCAUUGGUGU	Prr36, Zfp498, Cinp, Laptm5,Nipal3, Poc5, Ctdsp1, Gla
	rno-miR-324-3p	CCACUGCCCCAGGUGCUGCUGG	Pdha1, Baz2a, Nipal3, Enc1, Gypc, Map2, Cytip, Tcf23
rno-miR-448	rno-miR-448-5p	AACAUCCUGCAUAGUGCUGCCA	Atp6ap2, Bcor, Otc, Sytl5, Srpx, Xk, Cybb, Dynlt3
	rno-miR-448-3p	UUGCAUAUGUAGGAUGUCCCA	Bcor, Srpx, Tbc1d25, Rbm3,Ftsj1, Wdr13, , Gata1,Pqbp1
rno-miR-375	rno-miR-375-5p	GCGACGAGCCCCUCGCACAAACC	Mrm2, Sfn,Usp48, Fgf1, Rsad2, C5ar1, Grin2d, Gprin3
	rno-miR-375-3p	UUUGUUCGUUCGGCUCGCGUGA	Mark3, Scamp1, Nme5,Cys1, Asap2, Nr1h2,Gsg2, Relt
rno-miR-702	rno-miR-702-5p	GUGAGUGGGGUGGUUGGCAUG	Akap4, Cinp,Traf3, Stpg1, Lypla2, Kazn, Pla2g2d, Wdr41,
	rno-miR-702-3p	UGCCCACCCUUUACCCCACUCCA	Xk,Tsr2, Ephb4, Cdk5r2, Arl13a, Zadh2, Rce1, Snx32,
rno-miR-541	rno-miR-541-5p	AAGGGAUUCUGAUGUUGGUCACAC U	Syp, Pan3, Usp48, Shtn1, Smn1, Mat1a, Tnfsf18, Park7
	rno-miR-541-3p	AGUGGCGAACACAGAAUCCAUAC	Cpsf2, Cldn2, Atf6, Pcp4l1, Ptpn13, Rhot1, Coa4, Ptprr
rno-mir-218b	CAUGGUUAGAUCAAGCACAA		SMCP,POLR2G, TEX14, H2AFZ, ERI2, TRPV5, PIGW, PPP1R14C, SGK494, MBNL3

Table S1. The sequence and target gene of differentially expressed miRNAs
